# Data set for characterization of the glycosylation status of hepatic glycoproteins in mice fed a low-carbohydrate ketogenic diet

**DOI:** 10.1016/j.dib.2019.104604

**Published:** 2019-10-02

**Authors:** Tetsuya Okuda

**Affiliations:** Bio-Design Research Group, Bioproduction Research Institute, National Institute of Advanced Industrial Science and Technology (AIST), Central 6, 1-1-1 Higashi, Tsukuba, Ibaraki, 305-8566, Japan

**Keywords:** Ketogenic diet, Low-carbohydrate diet, Glycoprotein, Glycosphingolipid, Liver, *ob/ob*

## Abstract

The data presented herein pertain to a research article entitled “A low-carbohydrate ketogenic diet promotes ganglioside synthesis via the transcriptional regulation of ganglioside metabolism-related genes” [1]. The present article provides additional structural analysis data for the characterization of hepatic glycoproteins in mice fed a low-carbohydrate ketogenic diet (LCKD). Analysis of hepatic glycoproteins by enzyme-linked assay using the lectins UEA-I, ConA, LCA, and WGA showed that the LCKD decreased mature forms of complex-type glycans but increased immature forms of glycans on glycoproteins. An enzyme-linked immunosorbent assay using an anti–α2,6-sialyl LacNAc antibody also supported this result, indicating that dietary carbohydrate restriction results in aberrant glycosylation of tissue glycoproteins. These structural alterations of hepatic glycoproteins were not correlated with the expression levels of glycosyltransferase genes but were correlated with down-regulated expression of the *Gale* gene, which encodes a rate-limiting enzyme for the synthesis of sugar nucleotide donors for protein glycosylation in the liver. This property differed from glycosphingolipid metabolism in the liver of LCKD-fed mice.

**Table:** Specifications Table

**Subject**	Nutritional science, metabolism
**Specific subject area**	Low-carbohydrate ketogenic diet (LCKD), glycoprotein, glycosphingolipid
**Type of data**	Graph and Table
**How data were acquired**	Enzyme-linked lectin/immunosorbent assays, real-time PCR, HPLC
**Data format**	Raw and analyzed
**Parameters for data collection**	Mice fed a LCKD
**Description of data collection**	Five-week-old female C57BL/6J and B6.Cg-*Lep*^*ob*^/J mice were raised on either regular chow or a LCKD (F3666) for 7 weeks, after which tissue samples were collected. Glycoproteins were analyzed by enzyme-linked lectin/immunosorbent assays. Gene expression was analyzed by Agilent Expression Microarray and real-time PCR using SYBR Green I dye as the intercalator. Glycosphingolipids were analyzed by HPLC using a fluorescent labeling method [1].
**Data source location**	Bioproduction Research Institute, National Institute of Advanced Industrial Science and Technology (AIST), Central 6, Tsukuba, Japan
**Data accessibility**	With the article (Supplemental data) or a public repository Repository name: NCBI Gene Expression Omnibus (GEO) Data identification number: GSE115342 Direct URL to data: http://www.ncbi.nlm.nih.gov/geo/
**Related research article**	T. Okuda. A low-carbohydrate ketogenic diet promotes ganglioside synthesis via the transcriptional regulation of ganglioside metabolism-related genes. Scientific Reports. 9 (2019) 7627. https://doi.org/10.1038/s41598-019-43952-7 [1].

**Table:** 

**Value of the Data** • The data provide scientific evidence of the safety and effectiveness of the LCKD. • The data provide new insights into the effects of dietary interventions on the glycosylation status of tissue glycoproteins, which can be of value to researchers in related fields as well as health care workers. • LCKD-mediated alteration of the glycosylation status of tissue glycoproteins is a novel finding. • These data can be compared to other scientific data addressing the effects of the LCKD on various tissues. • The data and protocols provided here support other researchers investigating diet-induced metabolic abnormalities.

## 1 Data

**Fig. 1:**
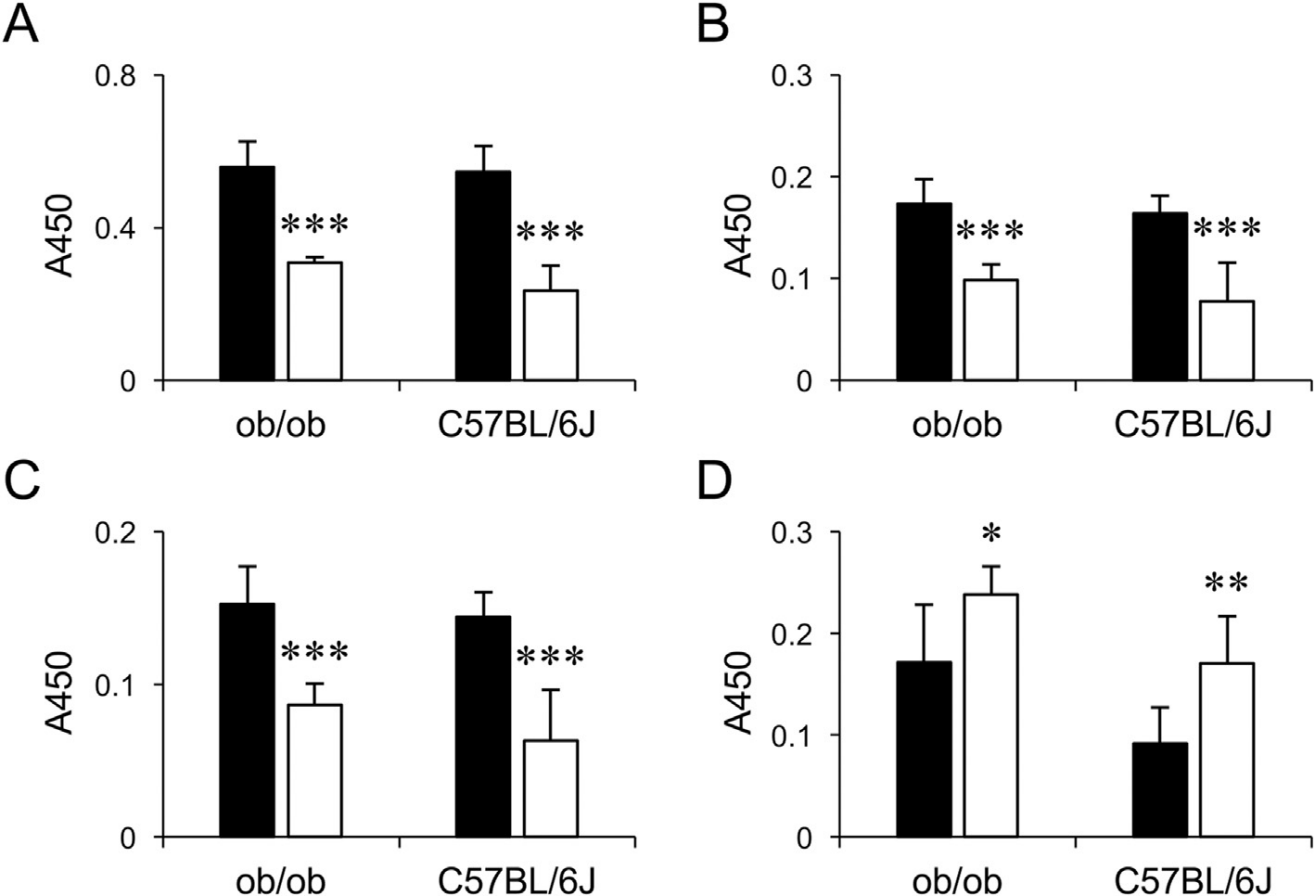
Analysis of hepatic glycoprotein glycans by enzyme-linked lectin assay. Individual extracts of liver proteins of chow-fed (closed bars) or LCKD-fed (open bars) *ob/ob* and C57BL/6J mice were analyzed by enzyme-linked lectin assay using UEA-I (A), Con A (B), LCA (C), and WGA (D). Mean ± S.D., n = 5–7. *P < 0.05, **P < 0.01, ***P < 0.001, chow-fed vs. LCKD-fed.

**Fig. 2:**
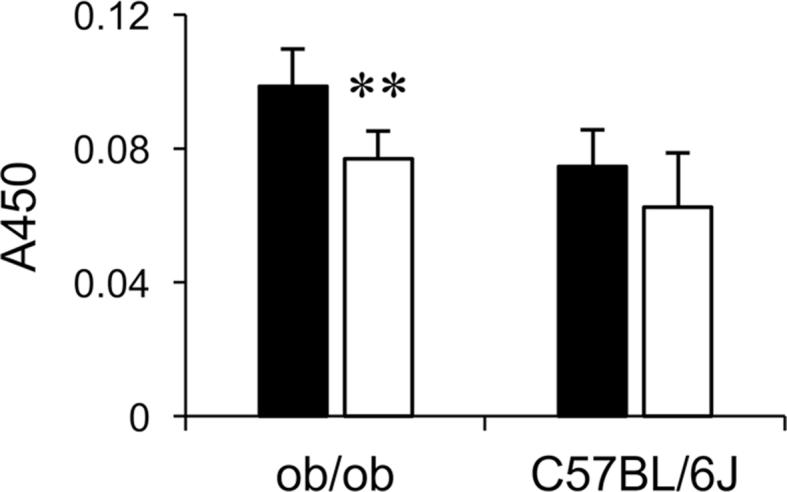
Analysis of hepatic glycoprotein glycans by enzyme-linked immunosorbent assay. Individual extracts of liver proteins of chow-fed (closed bars) or LCKD-fed (open bars) *ob/ob* and C57BL/6J mice were analyzed by enzyme-linked immunosorbent assay using an anti–α2,6-sialyl LacNAc antibody. Mean ± S.D., n = 5–7. **P < 0.01, chow-fed vs. LCKD-fed.

Fig. 1, Fig. 2 show data regarding the glycosylation status of hepatic glycoproteins in LCKD-fed C57BL/6J and B6.Cg-*Lep*^*ob*^/J (*ob/ob*) mice analyzed by enzyme-linked lectin/immunosorbent assays. *Ulex europaeus* agglutinin I (UEA-I) can be used to detect glycans containing terminal α-linked fucose residues, such as the blood group H(O) antigen [2,3]. Concanavalin A (Con A) can be used to detect α-linked mannose present as the terminal structure of high-mannose type *N*-glycans [3,4]. *Lens culinaris* agglutinin (LCA) can be used to detect α-linked fucose modification of the stem portion of *N*-glycans [3,5]. The FR9 monoclonal antibody (FR9) binds to α2,6-sialyl LacNAc, a common terminal structure of *N*-glycans [6]. Levels of these glycans, which are found in mature forms of glycoproteins, were uniformly decreased in the liver of LCKD-fed mice (Fig. 1, Fig. 2). In contrast, the reactivity of wheat germ agglutinin (WGA) against hepatic glycoproteins was higher in the LCKD-fed mice (Fig. 1D). As WGA can be used to detect the terminal *N*-acetylglucosamine structure of *N*-glycans that can be further glycosylated [3,7], these results indicate an increase in levels of immature forms of glycans on glycoproteins in the liver of LCKD-fed mice.

**Fig. 3:**
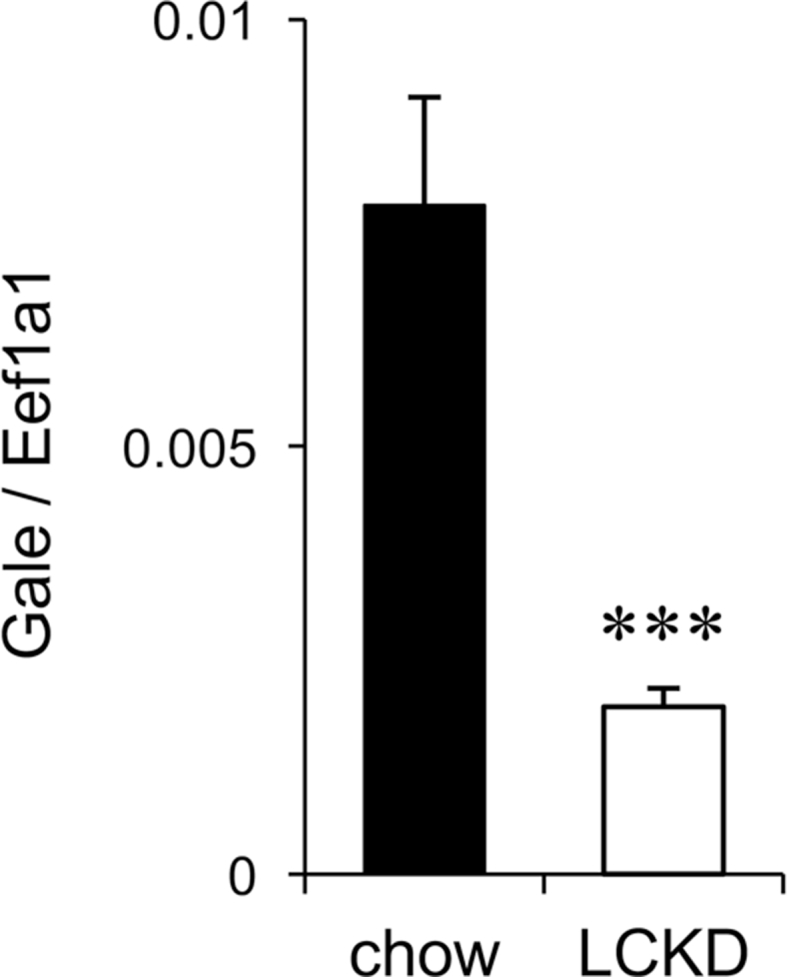
Real-time PCR analysis of *Gale* gene expression in the liver of *ob/ob* mice. Expression is shown as the ratio of *Gale* expression to expression of the internal standard (*Eef1a1*). Statistical significance was assessed using the two-tailed Student's *t*-test. ***P < 0.001, chow-fed vs. LCKD-fed. Closed bar, regular chow–fed mice; open bar, LCKD-fed mice. Mean ± S.D., (n = 5–6).

Fig. 3 shows the expression level of the UDP-galactose 4-epimerase gene (*Gale*), which encodes the rate-limiting enzyme for synthesis of sugar nucleotide donors for protein glycosylation in the liver [8], as determined by real-time PCR analysis. As LCKD-associated effects were clearly detected in *ob/ob* mice [1], we analyzed the hepatic expression of this gene in LCKD-fed *ob/ob* mice using an Agilent expression microarray. Data for all genes detected as specific signals were compared with data for mice fed regular chow (n = 3) and deposited in the Gene Expression Omnibus (GEO, accession number GSE115342). The major features of the results were published previously [1]. The microarray analysis revealed significant down-regulation (log^2^ ratio [LCKD/chow] of −1.88; *P* < 0.001) of *Gale* expression, which was confirmed in validation experiments using real-time PCR (Fig. 3) with a greater number of samples (n = 5–6). By contrast, the expression of almost all glycosyltransferase genes was up-regulated or unchanged in the LCKD-fed mice [1]. In other genes related to *Gale*, which involved in synthesis of sugar nucleotide donors, the galactose-1-phosphate uridyl transferase gene (*Galt*) also showed a slight decrease tendency in the expression level (log^2^ ratio [LCKD/chow] of −0.42; *P* < 0.05). These results indicate that dietary carbohydrate restriction and decreased synthesis of sugar nucleotide donors in LCKD-fed mice are correlated with alterations of the glycan structures of hepatic glycoproteins.

Table 1 shows the composition of glycosphingolipids in the liver of LCKD-fed mice. HPLC analysis showed that the LCKD increased levels of almost all glycosphingolipid species, unlike glycoprotein glycans. The results of structural analyses of glycosphingolipids in the liver of LCKD-fed *ob/ob* mice were published previously [1].

**Table 1:** Composition of glycosphingolipids in the liver of chow-fed and LCKD-fed mice.

Strain	GSL	Relative level			*P*-value
		chow	LCKD	Ratio (LCKD/chow)	
*ob/ob* (n = 3)	GM3-Ac	2167 ± 259	13098 ± 2981	6.04	0.0232*
	GM3-Gc	6993 ± 1027	25475 ± 3715	3.64	0.0011**
	GM2-Gc	110610 ± 11842	355805 ± 56026	3.22	0.0018**
	GM1-Ac	536 ± 121	2211 ± 298	4.13	0.0008***
	GM1-Gc	1066 ± 192	4189 ± 580	3.93	0.0009***
	GD1a	3624 ± 2956	5762 ± 779	1.59	0.2926
	Total	124997 ± 11586	406540 ± 60811	3.25	0.0014**
C57BL/6J (n = 4)	GM3-Ac	14425 ± 885	29321 ± 6231	2.03	0.0163*
	GM3-Gc	26669 ± 2621	35370 ± 6603	1.33	0.0498*
	GM2-Gc	539891 ± 36795	429724 ± 95956	0.80	0.0757
	GM1-Ac	1386 ± 260	3035 ± 521	2.19	0.0013**
	GM1-Gc	3924 ± 438	6352 ± 1319	1.62	0.0129*
	GD1a	8751 ± 1401	10200 ± 2346	1.17	0.3296
	Total	595046 ± 41119	514003 ± 112597	0.86	0.2251

Relative levels of glycosphingolipids in the liver were calculated based on the peak area (μV∙sec) of each glycosphingolipid detected in HPLC analysis, as described in the “Experimental design, materials, and methods” section. Values include previously reported data for *ob/ob* mice [1]. Mean ± S.D.; *P < 0.05, **P < 0.01, ***P < 0.001, chow-fed vs. LCKD-fed. Abbreviations: Ac, *N*-acetylneuraminic acid; Gc, *N*-glycolylneuraminic acid; GSL, glycosphingolipid; *ob/ob*, B6.Cg-*Lep*^*ob*^/J.

In both strains, body weight gain was similar in the chow- and LCKD-fed groups during the experimental period. In *ob/ob* mice, regular chow promoted significant steatosis associated with enlargement of the liver, but this pathology was suppressed in LCKD-fed mice. In contrast, the LCKD strongly promoted steatosis in C57BL/6J mice, although liver weight remained unchanged in chow- and LCKD-fed group. These results have been published elsewhere [1].

The raw data of figures are available in the Gene Expression Omnibus (GEO), repository, as accession number GSE115342 (https://www.ncbi.nlm.nih.gov/geo/query/acc.cgi?acc=GSE115342), or within this article (Supplemental data).

## 2 Experimental design, materials, and methods

### 2.1 Animals and dietary studies

Dietary studies using female *ob/ob* and C57BL/6J mice (Charles River Laboratories Japan, Yokohama, Japan) were conducted as reported previously [9,10]. CE-2 (CLEA Japan, Tokyo, Japan), composed of 58.2% carbohydrate, 12.6% fat, and 29.2% protein by calories, was used as regular chow. F3666 (Bio-Serv, Frenchtown, NJ), composed of 1.7% carbohydrate, 93.9% fat, and 4.4% protein by calories, was used as the LCKD. Five-week-old mice were raised on either regular chow or the LCKD for 7 weeks, after which samples were collected. The Committee for Experiments Involving Animals of the National Institute of Advanced Industrial Science and Technology approved all animal experiments.

### 2.2 Enzyme-linked lectin/immunosorbent assays

Protein extraction and *enzyme-linked immunosorbent assays* were conducted according to a previously reported method [11] using an α2,6-sialyl LacNAc antibody (FR9) [6]. Enzyme-linked lectin assays were performed by slightly modifying the enzyme-linked immunosorbent assay protocol. Briefly, 1 μg of hepatic protein was immobilized onto a 96-well microtiter plate (Nunc MaxiSorp F96; Thermo Fisher Scientific, Waltham, MA) and incubated at room temperature for 3 h with horseradish peroxidase (HRP)-linked lectins in 100 μl of blocking buffer (1% bovine serum albumin in phosphate-buffered saline [PBS]). After washing with 0.1% Tween-20 in PBS (PBST), HRP substrate (1-Step Ultra TMB-ELISA Substrate; Thermo Fisher Scientific) was used to detect lectin binding, and the results were measured as absorbance at 450 nm. Lectins were purchased from J-Oil Mills, Inc. (Tokyo, Japan).

### 2.3 Gene expression analysis

Preparation of total RNA and gene expression analysis were performed as reported previously [1]. Agilent expression microarray analysis for gene expression profiling in tissues was conducted by Takara Bio (Shiga, Japan). The resulting microarray data were analyzed using the Aqua microarray viewer and Aqua *t*-test (Takara Bio) and deposited in the NCBI GEO (http://www.ncbi.nlm.nih.gov/geo/) under accession number GSE115342. Relative quantification of target gene expression by real-time PCR was performed using a Light Cycler® 480 II system (Roche, Penzberg, Germany) with the following *Gale* gene-specific primers: forward, 5′-gtggttgccggacctaca; reverse, 5′-caccaccttgtacgggatct (accession number of the *Gale* gene: NM_178389, GenBank, https://www.ncbi.nlm.nih.gov/genbank/). Reactions were performed using a KAPA SYBR® FAST qPCR kit (KAPA Biosystems; Wilmington, MA) according to the manufacturer's instructions. Using this system, we first analyzed the expression levels of several housekeeping genes (*Actb*, *Gapdh*, *Eef1a1*) and found that *Eef1a1* was most stably expressed in the liver [1]. Thus, we used *Eef1a1* as the internal reference gene for subsequent real-time PCR analyses.

### 2.4 Glycosphingolipid extraction and HPLC analysis

Glycosphingolipid extraction and analysis by HPLC were performed as reported previously [1]. Briefly, glycosphingolipids were separated by Folch partitioning from total liver lipids, and their levels were determined by semi-quantitative HPLC using a glycosphingolipid fluorescent labeling method [1]. For fluorescent labeling, the ceramide moieties of purified gangliosides (corresponding to 30 mg of liver) were released by incubation in the presence of 6 mU of EGCase I (New England Biolabs, Ipswich, MA) at 37 °C for 16 h, and the reductive end of the oligosaccharide was fluorescently labeled using anthranilic acid (2-AA; Sigma-Aldrich, St. Louis, MO, USA) by incubation in 80 μl of labeling mixture (45 mg/ml 2-AA, 40 mg/ml sodium acetate trihydrate, 20 mg/ml boric acid, and 45 mg/ml sodium cyanoborohydride in methanol) at 80 °C for 1 h. The 2-AA–labeled oligosaccharides were analyzed using a TSK gel-amide 80 column (Tosoh, Tokyo, Japan) with an LC-2000 Plus HPLC system (JASCO, Tokyo, Japan). Relative levels of glycosphingolipids were calculated based on the peak area (μV∙sec) of each 2-AA oligosaccharide detected by the fluorescence detector of the HPLC system.

### 2.5 Statistical analysis

After determination of variance by the *F*-test, statistical significance was determined using the two-tailed Student's *t*-test, with statistical significance defined as follows: *P < 0.05, **P < 0.01, ***P < 0.001.
